# Retraction: Rbm46 regulates mouse embryonic stem cell differentiation by targeting *β-Catenin* mRNA for degradation

**DOI:** 10.1371/journal.pone.0259919

**Published:** 2021-11-09

**Authors:** The PLOS ONE Editors

Following the publication of this article [1], the authors CW, YC and LL contacted PLOS requesting the retraction of the article, stating that their email addresses were listed incorrectly on the article submission form, and that they were not aware of the article’s submission and had not approved the version to be published or agreed to be held accountable for all aspects of the work.

In reviewing this issue, additional concerns were raised regarding the results presented in Figs 1, 2, 3, 4, and 5. Specifically,

- Data are repeated across figure panels in Figs 1–4: Fig 1A and 2E report the same Rbm46 and Gapdh RT-PCR results; Fig 1A and 4A report the same Rbm46 and Beta-Actin western blot results; and Fig 3A and 3F report the same Rbm46 and Gapdh RT-PCR results. In each case, the first author noted that the repeated panels represent the same experiment but different terminology was used in figure labels.
- The tumour sizes reported in Figs 2 and 3 exceed sizes commonly accepted for mouse tumour studies. In response to the journal’s queries the first author clarified that whereas the Methods section mentions tumour measurements (“tumour growth was measured with a calliper [sic] every 3–5 days”) tumour size data were not collected over the course of the experiment but instead tumour weight data was only collected after animals had been euthanised.
- In Fig 5C, there appear to be abrupt discontinuities around the positive signal areas in the PABPC1 and Merge panels when levels are adjusted. The first author stated that no adjustments were made to the panels presented in Fig 5C, but the surrounding (background) areas of the published panels do not match the underlying data provided for the PABPC1 and Merge panels. This does not appear to impact the overall results reported in the figure.

The first author has provided individual level data for the results presented in the study but indicated that the raw uncropped blots underlying the published RT-PCR and western blot results are no longer available.

The authorship issues discussed above indicate that this submission did not comply with the PLOS Authorship policy. In light of this issue, the concerns regarding the reported mouse tumour sizes, and the concern about data reporting in Fig 5C, the *PLOS ONE* Editors retract this article.

LL and CW agreed with the retraction. LZ, YC, and SZ did not comment on the editorial decision. LL apologises for the issues with the published article.
